# mTORC1 signalling mediates PI3K-dependent large lipid droplet accumulation in *Drosophila* ovarian nurse cells

**DOI:** 10.1242/bio.022210

**Published:** 2017-03-16

**Authors:** Lawrence B. Mensah, Deborah C. I. Goberdhan, Clive Wilson

**Affiliations:** 1 Department of Physiology, Anatomy and Genetics, University of Oxford, Le Gros Clark Building, South Parks Road, Oxford OX1 3QX, UK; 2 The Koch Institute for Integrative Cancer Research, Massachusetts Institute of Technology, 500 Main Street, Cambridge, MA 02139, USA; 3 Department of Chemical Engineering, Massachusetts Institute of Technology, 500 Main Street, Cambridge, MA 02139, USA

**Keywords:** Obesity, Triacylglycerol, Tsc1, Tsc2, Akt, Insulin

## Abstract

Insulin and insulin-like growth factor signalling (IIS), which is primarily mediated by the PI3-kinase (PI3K)/PTEN/Akt kinase signalling cassette, is a highly evolutionarily conserved pathway involved in co-ordinating growth, development, ageing and nutrient homeostasis with dietary intake. It controls transcriptional regulators, in addition to promoting signalling by mechanistic target of rapamycin (mTOR) complex 1 (mTORC1), which stimulates biosynthesis of proteins and other macromolecules, and drives organismal growth. Previous studies in nutrient-storing germline nurse cells of the *Drosophila* ovary showed that a cytoplasmic pool of activated phosphorylated Akt (pAkt) controlled by *Pten*, an antagonist of IIS, cell-autonomously regulates accumulation of large lipid droplets in these cells at late stages of oogenesis. Here, we show that the large lipid droplet phenotype induced by *Pten* mutation is strongly suppressed when *mTor* function is removed. Furthermore, nurse cells lacking either *Tsc1* or *Tsc2*, which negatively regulate mTORC1 activity, also accumulate large lipid droplets via a mechanism involving *Rheb*, the downstream G-protein target of TSC2, which positively regulates mTORC1. We conclude that elevated IIS/mTORC1 signalling is both necessary and sufficient to induce large lipid droplet formation in late-stage nurse cells, suggesting roles for this pathway in aspects of lipid droplet biogenesis, in addition to control of lipid metabolism.

## Abstract (precis)

**Summary:** Signalling by the nutrient-sensitive mTORC1 pathway mediates the effects of elevated insulin-dependent PI3K on large lipid droplet formation in nutrient-storing nurse cells of the *Drosophila* ovary.

## INTRODUCTION

Proper control of the nutrient-regulated insulin and insulin-like growth factor signalling (IIS) cascade is essential in co-ordinating many basic cellular processes including cell proliferation, growth, nutrient homeostasis, lipid synthesis and longevity (Goberdhan and Wilson, 2003a; Soultoukis and Partridge, 2016). A key target pathway of insulin and insulin-like growth factors in IIS is the Class I phosphatidylinositol 3-kinase (PI3K)/phosphatase and tensin homologue (PTEN)/Akt kinase (also known as protein kinase B) signalling cassette. Defects in IIS have been linked to several major human diseases including many cancers, obesity, age-related cardiovascular disease and neurodegenerative disorders (Berryman et al., 2008, 2013). It also impacts several cell biological functions beyond metabolic biochemistry, such as the maintenance of mitochondrial integrity in *Drosophila* (Mensah et al., 2015).

IIS activation involves binding of insulin-like molecules to receptor tyrosine kinases [the Insulin Receptor (InR) in *Drosophila*] (Chen et al., 1996), which phosphorylate insulin receptor substrate (IRS) adaptor proteins (Chico in *Drosophila*) (Böhni et al., 1999) that in turn recruit and activate heterodimeric PI3K. PI3K catalyses the formation of phosphatidylinositol 3,4,5-trisphosphate [PtdIns(3,4,5)P_3_] from phosphatidylinositol 4,5-bisphosphate [PtdIns(4,5)P_2_]. PtdIns(3,4,5)P_3_ acts as a lipid second messenger that recruits the PH-domain-containing Akt protein kinase (Akt1 in *Drosophila*) (Staveley et al., 1998) to the plasma membrane, where it is activated by phosphorylation (Downward, 1998). The tumour suppressor protein PTEN is a lipid phosphatase that functions antagonistically to PI3K. *Pten* is frequently mutated or lost in cancer (Goberdhan and Wilson, 2003b; Leslie and Longy, 2016) and plays a key role in flies in restricting cell growth via Akt signalling (Gao et al., 2000; Goberdhan et al., 1999).

One major evolutionarily conserved family of molecules directly controlled by Akt consists of the FOXO transcription factors (FOXO1, -3, -4 and -6 in mammals; there is a single FOXO homologue in *Drosophila*) (Jünger et al., 2003; Webb and Brunet, 2014), which are inhibited by IIS. Activated Akt also indirectly stimulates the nutrient-sensitive mechanistic target of rapamycin complex 1 (mTORC1). One mechanism by which this is achieved involves phosphorylation of the tumour suppressor protein, tuberous sclerosis complex 2 (TSC2) within the TSC1/TSC2/TBC1D7 trimeric TSC complex. This releases TSC's inhibition of the G-protein Rheb (Ras homology enriched in brain), a positive regulator of mTORC1 (Dibble and Cantley, 2015).

mTORC1, in turn, affects several transcription factor targets, but also has a major effect on mRNA translation, driving cell growth and modulating nutrient homeostasis, lipid biogenesis and other metabolic events (Goberdhan et al., 2016). Hyperactivation of mTORC1 signalling produces tissue overgrowth in flies (Gao and Pan, 2001) and is commonly associated with tumorigenesis in humans. For example, loss-of-function of either *TSC1* or *TSC2* results in autosomal dominant tuberous sclerosis (TSC) (Astrinidis and Henske, 2005), characterized by formation of benign tumours (containing homozygous mutant cells) called hamartomas in multiple organs including the brain and kidneys (Arbiser et al., 2002). Cell proliferation in this disease is elevated, but some mutant cells also begin to store lipid by accumulating large lipid droplets (Astrinidis and Henske, 2005).

Lipid droplets (LDs) are evolutionarily conserved intracellular organelles with complex biological characteristics and functions in higher organisms (Beller et al., 2010; Guo et al., 2009). LDs in both white and brown adipocytes consist mainly of triacylglycerol (TAG) and cholesteryl esters. Although *Drosophila* does not have adipose tissue, it does have a related tissue-type, the fat body, for TAG storage (Kuhnlein, 2011). High levels of TAGs are also stored as small LDs in *Drosophila* stage 10 nurse cells of maturing egg chambers during oogenesis. At later stages, these LDs, together with other maternal factors, are pumped into the oocyte prior to ovulation and subsequent fertilisation, leading to development of the embryo (Li et al., 2012; McLaughlin and Bratu, 2015). The size of mature LDs varies depending on species and cell type. For example, LDs typically range from 100 µm diameter in adipocytes to ≤1-5 µm in *Drosophila* ovaries and 0.2-0.4 µm in normal yeast (Yang et al., 2012).

LDs are formed in the endoplasmic reticulum (Wilfling et al., 2013). They grow in size via a range of mechanisms, including LD fusion, lipid ester transfer and *de novo* synthesis *in situ* (reviewed in Ohsaki et al., 2014; Yang et al., 2012). Lipolysis of LDs is controlled by lipases, including hormone-sensitive lipase, which is regulated positively and negatively by β-adrenergic and insulin signalling, respectively, via effects on cAMP levels in adipose tissue (Lampidonis et al., 2011). IIS and mTORC1 signalling also affect the activity of Lipin, which positively regulates enzymes involved in TAG synthesis (Schmitt et al., 2015).

In *Drosophila*, we have found that elevation of IIS through loss of *Pten* function results in misregulation of lipid storage in nurse cells at late stages of oogenesis, leading to cell-autonomous accumulation of large lipid droplets (LLDs), but not in ovarian follicle cells (Vereshchagina and Wilson, 2006). This effect seems to be mediated by a subcellular pool of cytoplasmic pAkt1, which interacts with Widerborst (Wdb), one of the B’ regulatory subunits of protein phosphatase 2A (PP2A-B’) that binds to Akt1 (Fischer et al., 2016). Wdb normally keeps levels of cytoplasmic activated pAkt1 in check via a negative feedback loop (Vereshchagina et al., 2008). This effect of elevated germline IIS specifically in late-stage nurse cells is in sharp contrast to the effects of reduced germline IIS/mTORC1 during early oogenesis, which inhibits germline stem cell proliferation (Drummond-Barbosa and Spradling, 2001; LaFever et al., 2010) and can lead to developmental arrest in early or mid-oogenesis (Pritchett and McCall, 2012).

In this report, we investigate what downstream target pathways of Akt1 are involved in regulating LD size in nurse cells. Using genetic epistasis approaches in mutant germline nurse cell clones, we show that mTOR is required to produce the LLD phenotype seen in *Pten* mutant cells. Furthermore, loss of *Tsc1* or *Tsc2* can induce a *Pten*-like LLD phenotype, which is suppressed by *Rheb* loss-of-function. We conclude that in nurse cells, mTORC1 signalling plays a major role in mediating IIS-dependent LLD formation, an effect that might be related to lipid storage defects seen in patients with hamartomatous disease caused by loss of TSC function.

## RESULTS

### Analysis of lipid droplet phenotypes in *Drosophila* nurse cells

**Fig. 1.**
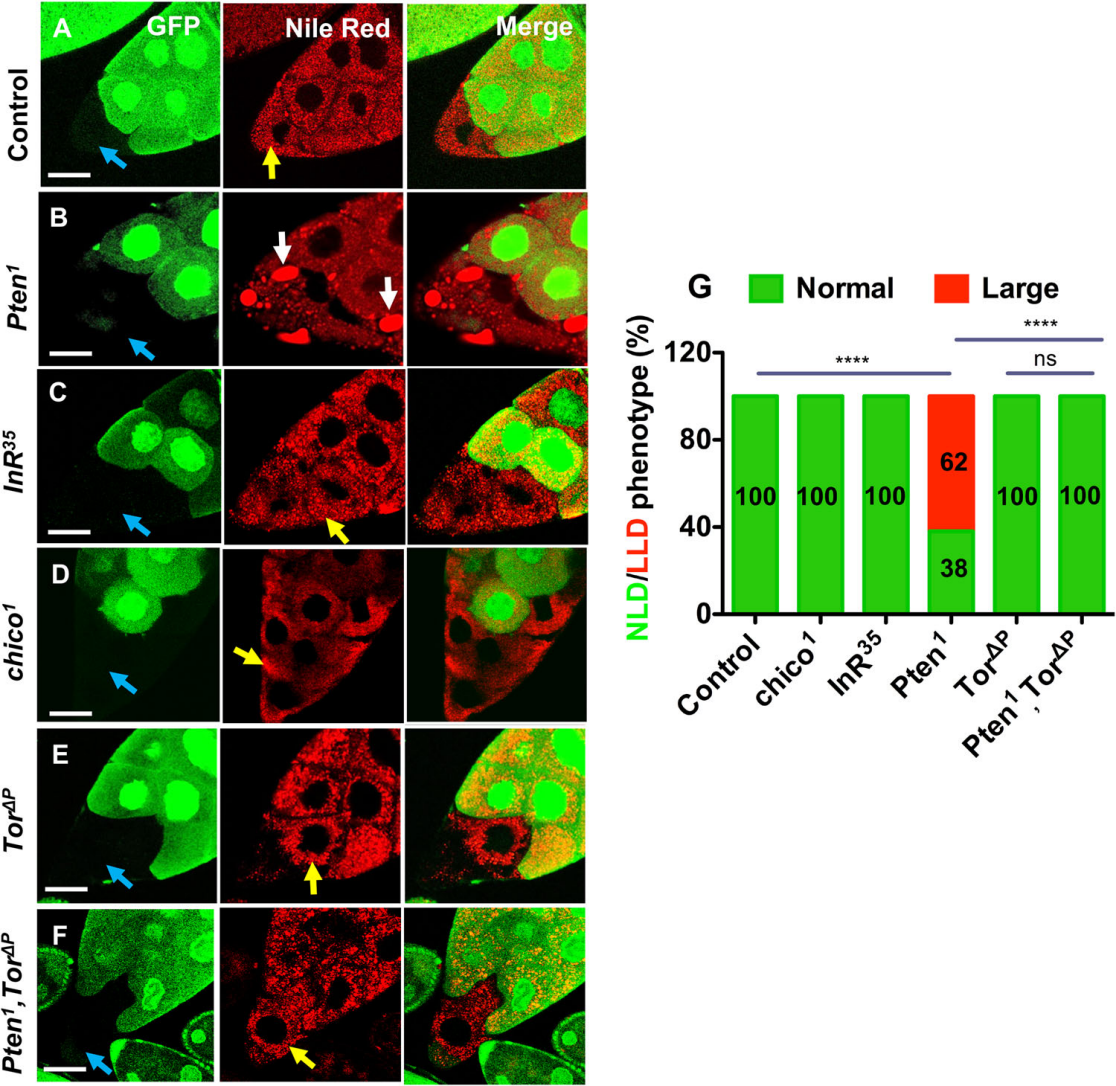
**Loss of *Pten* leads to *Tor*-dependent accumulation of LLDs in nurse cells.** (A-F) Ovaries containing mutant nurse cell clones (non-GFP-labelled; shown with blue arrow) were stained with lipid-soluble Nile Red dye to visualise lipid droplets. *Pten^1^* mutant cells contain large lipid droplets of variable size (white arrows in B). By contrast, much smaller lipid droplets are observed throughout the cytoplasm in wild-type control cells, and *InR^35^*, *chico^1^, Tor*^Δ*P*^ and *Pten^1^,Tor*^Δ*P*^ mutant nurse cells (yellow arrows in A,C,D,E,F). The lipid droplet phenotype for each mutant cell was classified as either ‘normal’ lipid droplet (NLD) or large lipid droplet (LLD) for each genotype as shown in the stacked bar chart (G), presenting the percentage of cells with NLD and LLD phenotypes. Genotypes and number of egg chamber clones and cells analysed: (A) *y w^1118^ hsp70-flp^122^/w^1118^; FRT40A/P[w^+^, Ubi-GFP] FRT40A* [total egg chamber clones (*N*)=30; number of cells (*n*)=48]; (B) *y w^1118^ hsp70-flp^122^/w^1118^; Pten^1^ FRT40A /P[w^+^, Ubi-GFP] FRT40A* (*N*=65; *n*=102);(C) *y w^1118^ hsp70-flp^122^/ w^1118^; InR^35^ FRT82B /P[w^+^, Ubi-GFP] FRT82B* (*N*=45; *n*=88); (D) *y w^1118^ hsp70-flp^122^/w^1118^; chico^1^ FRT40A/P[w^+^, Ubi-GFP] FRT40A* (*N*=40; *n*=72)*;* (E) *y w^1118^ hsp70-flp^122^/w^1118^;Tor*^Δ*P*^ *FRT40A/P[w^+^, Ubi-GFP] FRT40A* (*N*=58; *n*=87); (F) *y w^1118^ hsp70-flp^122^/w^1118^; Pten^1^,Tor*^Δ*P*^ *FRT40A/P[w^+^;Ubi-GFP] FRT40A* (*N*=52; *n*=107). (G) Data were analysed using Fisher's exact test. Statistically significant differences were observed between *Pten^1^* and all other genotypes, including control (wild type) and *Pten^1^,Tor*^Δ*P*^*.* (****P≤0.0001). All egg chamber images are at stage 10 of oogenesis. Scale bars: 40 μm.

To characterise in more detail the LD phenotypes observed in nurse cells when IIS is hyperactivated, we generated homozygous mutant clones (Fig. 1A-F) using the FLP/FRT system in adult females (Xu and Rubin, 1993), as previously reported (Vereshchagina et al., 2008; Vereshchagina and Wilson, 2006). Importantly, we did not employ the dominant female sterile technique for these experiments (St Johnston, 2002), where clones are induced in larvae. With this approach, several of the mutants that we used in this study produce abnormal egg chambers (e.g. Pritchett and McCall, 2012), which often do not develop to the late stages when changes in lipid droplet size are clearly visible. Since the cytoplasm of nurse cells is linked by ring canals, our nurse cell clone approach may mask the effect of some mutations if wild-type gene product diffuses into mutant cells via the ring canals. However, we had already found that this is not observed for *Pten* or *wdb* mutant clones or for the GFP marker used to distinguish clones (Vereshchagina and Wilson, 2006; Vereshchagina et al., 2008). Many of the other mutants described here also seem to exhibit cell-autonomous effects in nurse cells.

We analysed the size of LDs, stained with lipid-soluble Nile Red, in both mutant and control cells (Fig. 1G). LDs were classified as ‘Normal’ (NLDs) when they were in the range of 0-5 µm in diameter, and ‘Large’ (LLDs) when greater than 5 µm in diameter. All wild-type nurse cells contained only NLDs (Fig. 1A).

By contrast, in the majority of cells homozygous mutant for the *Pten* allele *Pten^1^*, which genetically behaves like a null allele (Goberdhan et al., 1999), at least one LLD was observed (Fig. 1B). We found that 62% of nurse cells homozygous for *Pten^1^* exhibited an LLD phenotype of this kind (Fig. 1G). Previously, we reported that 90% of *Pten^1^* clones contained enlarged lipid droplets (Vereshchagina and Wilson, 2006), which broadly mirrors our findings here, since many clones contain 1-3 mutant nurse cells. Importantly, mutant nurse cells at this and later stages of oogenesis, prior to cytoplasmic dumping into the oocyte, do not show any nuclear fragmentation, indicating that the lipid accumulation phenotype is not caused by apoptosis (Vereshchagina and Wilson, 2006). The strong loss-of-function *InR* allele, *InR^35^* (Fernandez et al., 1995), and the null *chico* allele, *chico^1^* (Böhni et al., 1999), which both reduce levels of IIS, had no detectable effect on LD size compared to controls (Fig. 1C,D,G).

### Induction of the LLD phenotype by hyperactivated IIS requires *Tor*

As a first assessment of whether increased mTORC1 signalling might mediate the LLD phenotype seen in *Pten* mutant clones, *Pten^1^* and null *Tor*^Δ*P*^ (Zhang et al., 2000) mutations were combined and double mutant homozygous clones analysed. In clones mutant for *Tor*^Δ*P*^ alone, no LLDs were observed and cells accumulated normal-sized LDs similar to control nurse cells (Fig. 1A,E). In the *Pten^1^*, *Tor*^Δ*P*^ double mutant (Fig. 1F), no LLDs were observed, representing a highly significant and strong suppression of the *Pten^1^* mutant phenotype (Fig. 1G). Loss of *Tor* is predicted to affect both mTORC1 signalling and mTORC2, an important positive regulator of Akt1 function (Hietakangas and Cohen, 2007). Therefore, although the suppression of the *Pten^1^* mutant phenotype by loss of *Tor* function is consistent with mTORC1 involvement in the IIS-dependent LLD phenotype, we could not exclude the possibility that the effect is also associated with reduction in mTORC2 activity.

### Loss of TSC function also induces LLD formation in nurse cells via its effects on Rheb/mTORC1 signalling

**Fig. 2.**
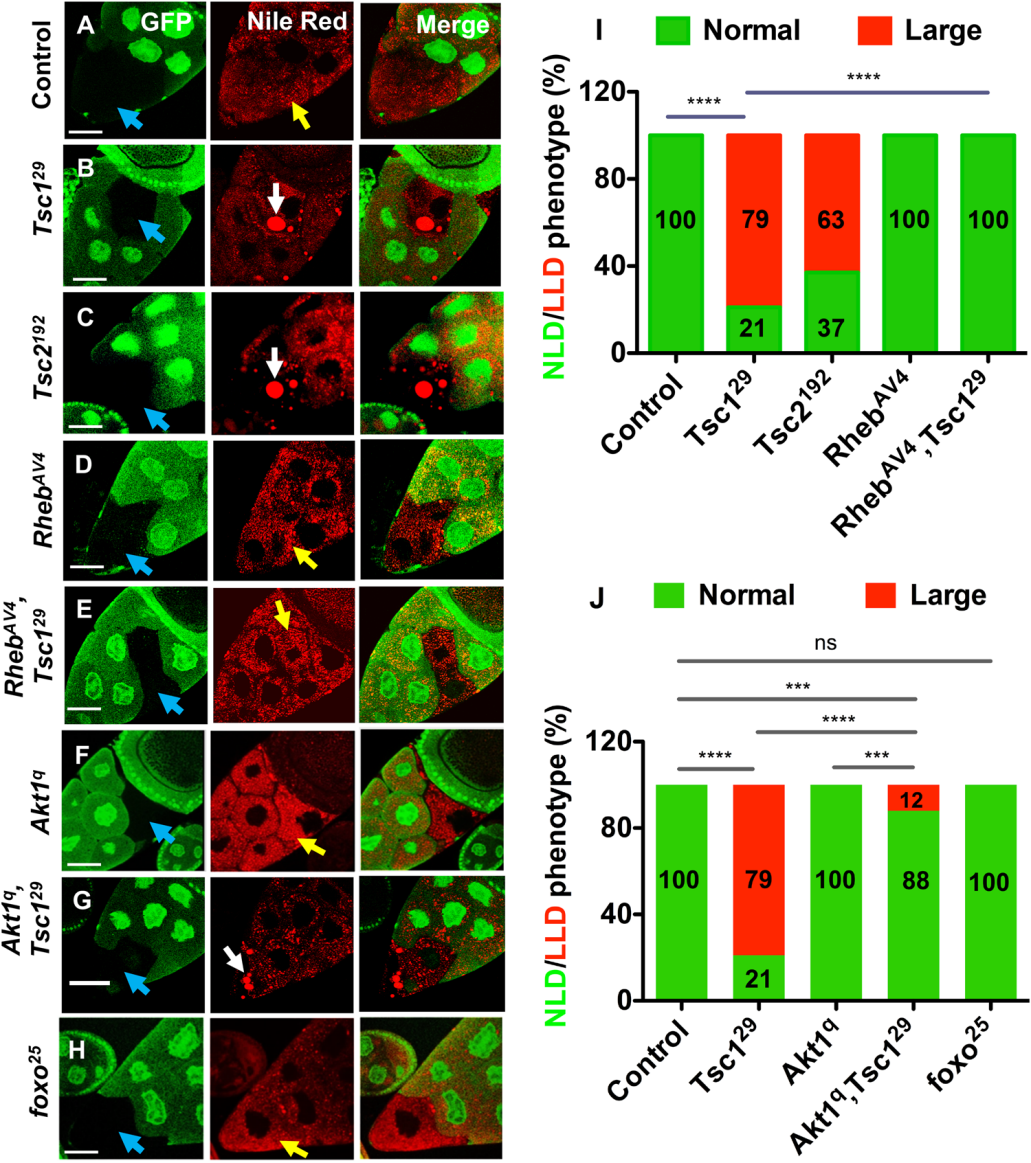
**Loss of *Tsc1* or *Tsc2* leads to Rheb-dependent accumulation of LLDs in nurse cells.** (A-H) Ovaries containing mutant nurse cell clones (non-GFP-labelled; blue arrows) were stained with lipid-soluble Nile Red dye. A normal lipid droplet phenotype is seen in wild-type, *Rheb^AV4^*, *Rheb^AV4^ Tsc1^29^*, *Akt1^q^* and *foxo^25^* clones (yellow arrows in A,D,E,F,H). By contrast, *Tsc1^29^* and *Tsc2^192^* mutant cells frequently contain large lipid droplets of variable size, some as large as 15μm in diameter (white arrows in B,C). Only a small proportion of *Akt1^q^ Tsc1^29^* mutant nurse cells contain LLDs (white arrow in G), although a further ∼40% have a phenotype with intermediate-sized droplets. (I,J) The size of lipid droplets in cells of all genotypes was analysed using Volocity and the percentage of cells with NLD and LLD phenotypes presented in the stacked bar charts. Genotypes and number of female flies dissected: (A) *y w^1118^ hsp70-flp^122^/w^1118^; FRT40A/P[w^+^, Ubi-GFP] FRT40A* [total egg chamber clones (*N*)=30; total number of cells (*n*)=48], (B) *y w^1118^ hsp70-flp^122^/w^1118^; Tsc1^29^ FRT82B/P[w^+^, Ubi-GFP] FRT82B* (*N*=55; *n*=103); (C) *y w^1118^ hsp70-flp^122^/w^1118^; Tsc2^192^ FRT80B /P[w^+^, Ubi-GFP] FRT80B* (*N*=61; *n*=79); (D) *y w^1118^ hsp70-flp^122^/w^1118^; Rheb^AV4^ FRT82B /P[w^+^, Ubi-GFP] FRT82B* (*N*=57; *n*=138); (E) *y w^1118^ hsp70-flp^122^/w^1118^; Rheb^AV4^,Tsc1^29^ FRT82B/P[w^+^,Ubi-GFP] FRT82B* (*N*=42; *n*=67). (F) *yw^1118^ hsp70-flp^122^/w^1118^, Akt1^1q^ FRT82B/P [w^+^, Ubi-GFP] FRT82B* (*N*=45; *n*=68); (G) *y w^1118^ hsp70-flp^122^/w^1118^ ; Akt1^1q^ ,Tsc1^29^ FRT82B/ P[w^+^,Ubi-GFP] FRT82B* (*N*=51; *n*=112); (H) *y w^1118^ hsp70-flp^122^/w^1118^ ; foxo^25^ FRT82B/ P[w^+^, Ubi-GFP] FRT82B* (*N*=65; *n*=77). Statistically significant differences were observed between control (wild type) and single mutants *Tsc1^29^* and *Tsc2^192^*, and also between *Tsc1^29^* and double mutant *Rheb^AV4^,Tsc1^29^.* (J) The *Tsc1^29^* LLD phenotype is strongly, but not completely, suppressed by *Akt1^q^*, while *foxo^25^* has no effect on LLD formation compared with control cells. (***P≤0.001, ****P≤0.0001). Scale bars: 40 μm.

To examine whether increased mTORC1 signalling might itself induce LLD accumulation in nurse cells, we generated nurse cell clones mutant for null alleles of *Tsc1* and *Tsc2* (known as *gigas*), namely *Tsc1^29^* and *Tsc2^192^* (Gao and Pan, 2001), respectively. Unlike wild-type cells, 79% of *Tsc1^29^* (Fig. 2B) and 63% of *Tsc2^192^* (Fig. 2C) mutant cells contained LLDs (Fig. 2I). As we have found previously for *Pten* mutant clones, *Tsc* mutant clones showed no indication of nuclear fragmentation at stage 10 or 11 (Fig. S1), suggesting that the LLD phenotype is not caused by apoptosis. Other studies in which germline clones are induced during larval development (Pritchett and McCall, 2012) have indicated that reducing IIS/mTORC1 signalling throughout oogenesis leads to egg chambers arresting in early to mid-oogenesis. However, in our study, by inducing clones in adults and only culturing females for a further 24-36 h, analysed clones are induced at mid-oogenesis, and we see no evidence of abnormal egg chamber development or arrest with any mutant. Therefore, we conclude that loss of key antagonists of the mTORC1 pathway can produce lipid accumulation phenotypes similar to *Pten* loss-of-function in nurse cells, suggesting that mTORC1 signalling is important in mediating the lipid-associated phenotypes caused by hyperactivated IIS.

To confirm that the effects of blocking TSC activity on LLD formation are mediated by mTORC1 hyperactivation, the *Tsc1^29^* allele was combined with a strong loss-of-function mutation in *Rheb*, *Rheb^AV4^* (Patel et al., 2003). Rheb's activation of mTORC1 is inhibited by the TSC complex. As expected, we found that 100% of *Rheb^AV4^* mutant nurse cells exhibited no LLDs (Fig. 2D). When combined with *Tsc1^29^*, the *Tsc1*-dependent LLD phenotype was completely suppressed (Fig. 2E,I), indicating that Rheb is necessary for the formation of LLDs in *Tsc1* mutant nurse cells and strongly implicating hyperactivated mTORC1 signalling in LLD induction.

Akt1 regulates a number of downstream targets (Downward, 1998), which might also have a role in LLD biogenesis. We tested whether Akt1 activity is required for elevated mTORC1 signalling to induce LLD formation by combining loss-of-function alleles for *Akt1* and *Tsc1*. Nurse cells homozygous for a null *Akt1* allele, *Akt1^q^* (Staveley et al., 1998), did not exhibit a lipid storage defect (Fig. 2F). When combined with the *Tsc1^29^* allele, only 12% of homozygous mutant cells contained LLDs (Fig. 2G,J). This suggests that although elevated mTORC1 signalling can drive LLD formation, some upstream IIS signalling is required to permit this.

One possible explanation for this result is that the key transcription factor and inhibitory target of Akt1, Foxo, also plays a role in regulating LLD formation in nurse cells. To test this, we generated homozygous mutant clones for the null *foxo* allele, *foxo^25^* (Jünger et al., 2003). There was no lipid accumulation phenotype in these cells (Fig. 2H,J), suggesting that loss of *foxo* alone is insufficient to alter lipid droplet size in nurse cells.

## DISCUSSION

Several studies have revealed that both starvation and reducing IIS/mTORC1 signalling during different stages of oogenesis affect the survival and differentiation of germ line and somatic cells in the ovary (Burn et al., 2015; Drummond-Barbosa and Spradling, 2001; Pritchett and McCall, 2012). Our previous work has revealed that nutrient-storing nurse cells of the fly ovary, which are homozygous for loss-of-function alleles of the tumour suppressor *Pten*, a major antagonist of IIS, accumulate LLDs (Vereshchagina and Wilson, 2006). Here, we demonstrate that activating downstream mTORC1 signalling in nurse cells induces a similar cell type-specific phenotype, although genetic epistasis experiments suggest that to fully exhibit this phenotype, basal IIS may also be required.

### The roles of IIS/mTORC1 signalling in lipid droplet storage and *Drosophila* egg chamber development

Co-ordinated activity of the IIS and mTORC1 signalling pathways is essential for a number of important physiological and metabolic cellular functions (Goberdhan et al., 2016; Wilson et al., 2007), including the regulation of lipid storage in *Drosophila* nurse cells and other tissues, particularly the fat body. When IIS and mTORC1 signalling are active in the fed state, FOXO is excluded from the nucleus, cell growth is stimulated and LD formation is enhanced in the fat body, as it is in mammalian adipose tissue (see review in Baker and Thummel, 2007). Conversely, in the starved state, IIS and mTORC1 activity is decreased, resulting in FOXO translocation into the nucleus and expression of genes involved in lipolysis. In *Drosophila*, lipid droplet surface molecules called perilipins (LSD1 and LSD2), and the hormone-sensitive lipase and adipose triglyceride lipase homologues are involved in the regulation of lipid droplet size and number (Bi et al., 2012; Grönke et al., 2005). We have previously shown that increased IIS signalling in nurse cells can increase LSD2 expression (Vereshchagina et al., 2008), but other mechanisms are likely to be involved in the LLD phenotype. Indeed, our new data suggest that IIS and mTORC1 signalling may work together to induce the formation of LLDs in nurse cells.

A significant body of other work has highlighted several additional important roles for IIS/mTORC1 signalling in oogenesis. Aberrant signalling in either somatic follicle cells or the germline inhibits normal oogenesis (Burn et al., 2015; Barth et al., 2011; Drummond-Barbosa and Spradling, 2001; LaFever et al., 2010; Pritchett and McCall, 2012). Most notably, reducing IIS/mTORC1 in the germline inhibits stem cell proliferation (Drummond-Barbosa and Spradling, 2001; LaFever et al., 2010) and induces a developmental arrest in early to mid-oogenesis (Pritchett and McCall, 2012). Starvation induces programmed cell death in egg chambers at mid-oogenesis (Drummond-Barbosa and Spradling, 2001), a mechanism that saves metabolic resources. However, for many germline clones with reduced IIS/mTORC1 signalling, developmental arrest occurs without nurse cell nuclear fragmentation, although surrounding follicle cells appear to be lost (Pritchett and McCall, 2012).

To circumvent these early effects caused by inhibiting IIS/mTORC1 signalling in the germline, we employed a clonal approach in which clones are induced in adults and then analysed within 24-36 h. The stage 10 and 11 egg chambers containing mutant nurse cells that we analysed do not seem to arrest in development, perhaps because these mutant cells will have been produced around mid-oogenesis (Calvi et al., 1998), after the period when IIS/mTORC1 signalling appears to be critical for developmental progression (Pritchett and McCall, 2012).

Nurse cells begin to dump their contents, including lipid droplets (Teixeira et al., 2003), into the oocyte at stage 11. Since we observe the LLD phenotype in stage 10 egg chambers, it seems unlikely that this is caused by lipid droplets becoming trapped inside nurse cells. However, it becomes more difficult to score mutant nurse cells in egg chambers from stage 11 onwards, because of increased cytoplasmic exchange between these cells. Hence, although we see no premature nuclear fragmentation or obvious arrest of late-stage egg chambers in our experiments, we cannot exclude that very late-stage developmental defects do occur.

### Genetic manipulations that increase mTORC1 signalling induce LLD formation in nurse cells

To test whether activation of mTORC1 signalling is sufficient to produce LLDs, we genetically increased mTORC1 pathway activity by creating homozygous mutant clones of null *Tsc1* and *Tsc2* (Gao and Pan, 2001) alleles in nurse cells and in columnar follicle cells. The LLD phenotypes observed in nurse cells were as penetrant as those seen with *Pten* mutant clones (Vereshchagina et al., 2008). Importantly, the complete suppression of the *Tsc^129^*-mediated and *Pten^1^*-mediated LLD phenotypes by *Rheb* and *Tor* mutations respectively strongly supports our hypothesis that this phenotype requires elevated mTORC1 signalling.

We were more surprised by the genetic interactions between the *Tsc1^29^* and *Akt1^q^* alleles in mutant nurse cell clones. In the linear model of IIS/mTORC1 signalling, mTORC1 activation lies downstream of the PI3K/PTEN/Akt signalling cassette. In fact, molecules downstream of Akt are known to regulate cell-surface IIS through at least two negative-feedback loops involving mTORC1-regulated S6 kinase and FOXO (Goberdhan et al., 2005; Harrington et al., 2005; Puig and Tjian, 2005). We might have therefore expected *Tsc1* mutations to reduce Akt1 activity so that Akt1 mutations would not affect LLD formation. However, at least one genetic study suggests that the TSC complex can regulate mTORC2 activity and therefore act as an inhibitor of Akt1 (Natarajan et al., 2013). Thus, it remains possible that both Akt1 and mTORC1 signalling need to be elevated in nurse cells to generate LLDs. It will be interesting to explore this idea further in the future, particularly since the control of LLDs is associated with activated Akt1 in the cytosol (Vereshchagina and Wilson, 2006), which may be regulated differently to activated Akt1 recruited to the plasma membrane by increased PI3K signalling.

Although mutations in *Pten*, *Tsc1*, *Tsc2*, *Akt1*, *Rheb* and *Tor* all either generate an LLD phenotype or strongly suppress this phenotype in nurse cell clones, for *chico*, *InR* and *foxo* mutants, we did not observe any mutant phenotype. Based on analysis of other positive regulators of IIS/mTORC1 signalling, we would not have predicted that either *chico* or *InR* mutations would generate an LLD phenotype in clones induced at mid-oogenesis. However, using this approach, the lack of an LLD phenotype in *foxo* nurse cells could be explained by leakage of wild-type gene product from normal cells into mutant nurse cells through the ring canal. Therefore, we cannot exclude the possibility that FOXO is involved in the LLD phenotype, although our data suggest that activation of mTORC1 signalling is essential for nurse cell LLD induction by increased IIS.

### IIS/mTORC1 signalling, lipid droplet size and disease

Changes in LD phenotypes are often associated with human diseases, such as Type 2 diabetes, atherosclerosis, cancer and other lipodystrophies (Krahmer et al., 2013). A better understanding of the mechanisms by which IIS/mTORC1 signalling alters lipid accumulation might provide new insights into such disease links. For example, loss-of-function in human *TSC* genes can cause a rare benign renal tumour known as angiomyolipoma (AML) (Arbiser et al., 2002; Astrinidis and Henske, 2005). This tumour is characterised by a combination of increased proliferative tissue and generation of adipose tissue with considerable lipid accumulation that can be suppressed, but not fully blocked, by mTOR inhibitors (Budde et al., 2016; Siroky et al., 2012). Our data indicating a potential dual role for IIS and mTORC1 in nurse cell lipid droplet accumulation suggest that more than one TSC-dependent pathway may need to be inhibited in order to achieve complete therapeutic response.

Obesity is also a well-established predisposing factor in the acquisition of cellular insulin resistance and Type 2 diabetes (Haslam and James, 2005). Increased levels of circulating free fatty acids (FFAs) associated with obesity appear to be important in this link (Kovacs and Stumvoll, 2005). However, it is unclear whether other mechanisms are also involved or how reduced insulin sensitivity ultimately impacts on lipid storage. Our work has raised the possibility that cytoplasmic and cell surface pAkt may be independently controlled in some cell types, with the former specifically promoting lipid droplet size and storage, and also selectively suppressing insulin-dependent events at the cell surface. It will be interesting to investigate whether these different pAkt pools can affect each other's activity via the multiple feedback mechanisms in the IIS/mTORC1 pathway, and whether this might play a role in linking obesity and insulin resistance in diseases such as Type 2 diabetes.

## MATERIALS AND METHODS

### *Drosophila* stocks and generation of mosaic clones

Except where mentioned, all flies were obtained from the Bloomington Stock Centre (Indiana, USA). The FLP/FRT site-specific recombination system has been described (Xu and Rubin, 1993) and was used to generate clones in the fly germ line and somatic tissues. The following genotypes were used to generate homozygous mosaic clones in ovarian and follicular cells.

*yw^1118^ hsp70-flp^122^; [w+,Ubi-GFP FRT,52A] FRT40A /TM6B Hu,Tb* males, which produce leaky expression of flp even in the absence of heat shock (Britton et al., 2002) were crossed to females of the following genotypes: *w; Pten^1^ FRT40A/CyORoi*; *yw^1118^; Tor*^Δ*P*^ *FRT40A/CyO* (a gift from Neufeld, Minnesota, MN, USA) and *w^1118^; Pten^1^ Tor*^Δ*P*^ *FRT40A/CyO. w; chico^1^ FRT40A/CyO males* were crossed to females homozygous for *yw^1118^ hsp70-flp^122^; P[w^+^;Ubi-GFP^nls^S65T] FRT40A* (a gift from Bruce Edgar, Fred Hutchinson Cancer Research Center, Seattle, USA), which preferentially expresses GFP in the nucleus. Newly eclosed females carrying *hsp70-flp^122^* and the FRT40A chromosomes were selected for clonal analysis.

*yw^1118^ hsp70-flp^122^ (leaky); FRT82B [w+, Ubi-GFP FRT] /TM6B Hu,Tb* males were crossed to *yw^1118^; Tsc1^29^FRT82B /TM6B Hu,Tb* (gift from D. Pan, University of Texas Southwestern, *USA)*; *yw^1118^; Rheb^AV4^FRT82B /TM3 Sb* (gift from J. Lengyel, University of California, USA); *w P[w+,FRT2A]; Akt1^q^ FRT82B /TM3 Sb* (gift from B. Staveley, Memorial University of Newfoundland, Canada); *w^1118^; Rheb^AV4^ FRT82B Tsc1^29^/TM3 Sb* and *w; Akt1^q^ Tsc1^29^FRT82B /TM3 Sb. w; Inr^35^FRT82B /TM6 Hu* and *w; foxo^25^FRT82B /TM6 Hu, Tb* (gifts from Ernst Hafen,University of Zurich, Switzerland) males were crossed to females homozygous for *yw^1118^ hsp70-flp^122^ (leaky);FRT 82B[w,Ubi-GFP FRT].* The newly eclosed *hsp70-flp^122^* females carrying the FRT82B chromosomes were selected for clonal analysis.

*yw^1118^ hsp70-flp^122^; FRT80B [w+; Ubi-GFP FRT]/TM6 Hu,Tb* males were crossed to *w; Tsc2^192^ FRT 80B/TM6B Hu,Tb* females (a gift from D. Pan, University of Texas Southwestern, USA). Newly eclosed *y w^1118^ hsp70-flp^122^/w^1118^; Tsc2^192^ FRT80B /P[w+, Ubi-GFP] FRT80B* females were selected for clonal analysis.

### Apple juice-yeast broth enriched cornmeal food

To one litre of boiling water, 10.5 g of technical grade agar, 75.0 g of cornmeal (pre-mixed in a small volume of cold water), 31.5 g of dried yeast, 93.0 g of glucose, 8.6 g of sodium potassium tartrate, and 0.7 g of calcium chloride (pre-mixed in a small volume of hot-water) were added. The mixture was gently simmered and thoroughly mixed for approximately 20 min. 2.5 g of methyl-4-hydrobenzoate [nipagen] (pre-dissolved in 12 ml of ethanol) was added and stirred in. With the aid of a 100 vial (10×10) dispenser (Workshop, DPAG, University of Oxford, UK), 5-8 ml of fly food was aliquoted into 40 ml vials. 30-40 ml of food was aliquoted into 250 ml bottles. The food was allowed to cool and vials were plugged with cotton wool and stored at 4°C. To prepare yeast-enriched medium for fly culture, 2.5 g of dried yeast were dissolved in 40 ml of apple juice and fermented overnight at ambient temperature in the dark. This broth was then stored at 4°C. 2-3 drops of apple-juice-yeast broth was added to each vial of standard cornmeal food and allowed to air-dry prior to fly culture.

### Generation of homozygous mutant clones in the ovary

Female flies of the appropriate genotype were heat-shocked as previously described (Vereshchagina and Wilson, 2006) to induce mosaic homozygous clones in nurse cells and epithelial follicle cells. Briefly, crosses to generate females were cultured at 18°C on apple-juice-enriched food. Newly eclosed females of the appropriate genotype were collected and heat-shocked for 1 h at 37.5°C in a water bath. These flies were transferred together with male flies onto freshly prepared apple juice-enriched cornmeal food to induce more oogenesis and maintained at 25°C. Female flies were aged for 24-36 h depending on genotype in a 25°C incubator before ovary dissection*.*

### Lipid droplet staining

The abdomens of anaesthetized flies were removed using a scalpel blade and immediately transferred into cold 1×PBS, 0.1% Tween 20 (PBST). The ovarioles were carefully dissected and fixed in 0.8 ml 4% paraformaldehyde (in PBS, pH 7.4) for 30 min at ambient temperature. Ovaries were washed for 3×15 min in 1 ml 1×PBST, then stained with Nile Red for 20 min at ambient temperature [a 10 mg/ml solution of Nile Red dye in acetone was diluted 1:2500 in 1× PBST (4 µg/ml final concentration)] and incubated with ovaries for 20 min. DAPI (1.5 µg/ml) was subsequently used to stain nuclear material for 1 h at ambient temperatures. The ovaries were washed for 3×5 min in PBST after each staining. Ovaries were mounted on a microscope slide with 80% glycerol+2.5% propyl-gallate mountant (Sigma-Aldrich, Dorset, UK).

### Confocal microscopy and lipid droplet imaging

All fluorescent images were captured on a Carl Zeiss Axioplan 2 LSM 510 META laser confocal microscope. Nile Red-stained ovaries were first scanned with the 10× objective to identify mutant clones and then the 40× and 63× oil objective lenses were used to capture images. Nile Red fluorescent emission was imaged at 636 nm, GFP at 512 nm and DAPI at 461 nm. Images were imported and analysed with LSM 510 software and browser. Confocal image rotation, signal normalization, and merging of different channels were all carried out using Adobe Photoshop CS4 software.

### Analysis of lipid droplets by size and number

At least three independent experiments were performed for each genotype, from which Nile Red-stained mutant or control nurse cells were analysed for LD diameter. We measured the diameter of lipid droplets using two image analysis programmes. Zeiss LSM 510 Meta confocal microscope software was used to process images, and then these were imported into Volocity software (Improvision) to measure lipid droplet diameter. A hand-held image tracer was used to outline all lipid droplets per mutant cell to compute droplet size as previously described (Watanabe et al., 2010). LDs were classified as ‘Normal’ (NLDs) when they were in the range of 0-5 µm in diameter, and ‘Large’ (LLDs) when greater than 5 µm in diameter. All wild-type nurse cells were found to contain only NLDs. For other genotypes, we calculated the number of mutant cells containing one or more LLD, using a minimum of 30 females and more than 150 stage 10 egg chambers for each genotype (the number of cells analysed for each genotype is given in the legend). Fisher's exact test was used for statistical analysis of the two nominal variables, genotype and NLD/LLD phenotype; those pairs of genotypes, which appear to have significantly different proportions of NLD versus LLD cell phenotypes, are highlighted in the figures.
